# Single-cell gene expression and TCR profiling reveal age-related differences in recent thymic emigrants

**DOI:** 10.1016/j.isci.2026.115582

**Published:** 2026-04-01

**Authors:** Cybelle Tabilas, Vanessa Venturi, Connor Kean, Anastasia Minervina, Paul G. Thomas, Andrew Grimson, Jennifer K. Grenier, Miles P. Davenport, Norah L. Smith, Brian D. Rudd

**Affiliations:** 1 Department of Microbiology and Immunology, Cornell University, Ithaca, NY 14853, USA; 2 Kirby Institute for Infection and Immunity, UNSW Australia, Sydney, NSW 2052, Australia; 3 Department of Molecular Biology and Genetics, Cornell University, Ithaca, NY, USA; 4 Department of Host-Microbe Interactions, St. Jude Children’s Research Hospital, Memphis, TN, USA; 5 Genomics Facility, Biotechnology Resource Center, Cornell University, Ithaca, NY 14853, USA

**Keywords:** immunology, transcriptomics

## Abstract

Following thymic egress, CD8^+^ T cells undergo post-thymic maturation to transition from recent thymic emigrants (RTEs) to mature naive T cells. Although RTEs are phenotypically and functionally distinct from mature naive CD8^+^ T cells, most studies on RTEs have been performed in adults. As a result, little is known about the behavior of RTEs made in early life, which is when they are most abundant. Here, we used a fate mapping mouse model to compare neonatal and adult CD8^+^ RTEs and found that they exhibit distinct phenotypes and functions. Paired single-cell transcriptomics and T cell receptor (TCR) sequencing showed that neonatal RTEs exhibit a more effector-like gene expression profile than adult RTEs, and the most pronounced effector-gene bias was found in neonatal RTEs that utilize germline-encoded TCRs. Collectively, these data reveal how the RTE pool changes during development and how TCR usage contributes to phenotypic heterogeneity in the neonatal and adult RTE pools.

## Highlights

• The phenotype and function of CD8^+^ RTEs depend on when they are produced
• The neonatal CD8^+^ RTE pool contains more virtual memory cells
• All neonatal CD8^+^ RTEs display a more effector-like gene expression profile
• Post-thymic maturation is not a major driver of the neonatal CD8^+^ T cell phenotype

## Graphical abstract

**Figure:**
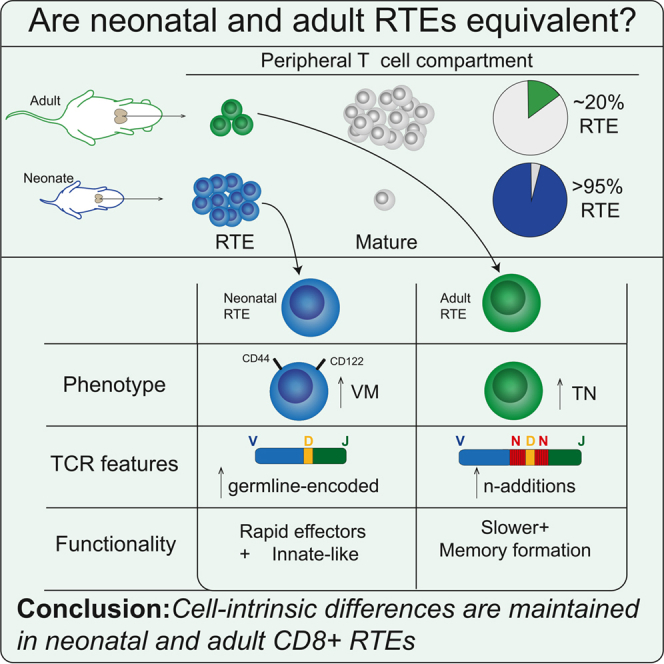

## Introduction

Previous work has shown that the phenotype and function of CD8^+^ T cells continue to evolve after they egress from the thymus.^1^^,^^2^^,^^3^^,^^4^^,^^5^ The subset of murine CD8^+^ T cells that are less than 3 weeks old, denoted as recent thymic emigrants (RTEs), is phenotypically and functionally distinct from its more mature counterparts.^6^^,^^7^ While RTEs make up a small percentage (∼10%–20%) of naive cells in adult mice, they account for nearly 100% of T cells in neonatal mice.^8^ Interestingly, the functional differences between RTEs and mature T cells in adult mice are reminiscent of the differences that have been previously described in naive CD8^+^ T cells from neonatal and adult mice. For example, both adult CD8^+^ RTEs and neonatal CD8^+^ T cells preferentially become short-lived effector cells during infection.^4^^,^^9^^,^^10^^,^^11^ Moreover, adult CD8^+^ RTEs and neonatal CD8^+^ T cells exhibit more innate-like functions, as evidenced by their innate-like receptor expression (complement receptors and NK cell receptors) and ability to deploy non-specific defense mechanisms more typically associated with innate cells.^12^^,^^13^^,^^14^^,^^15^^,^^16^^,^^17^^,^^18^^,^^19^ As a result, there is an assumption that neonatal CD8^+^ T cells are simply immature adult CD8^+^ T cells or adult CD8^+^ RTEs.

To test the theory that developmental changes in the CD8^+^ T cell response correspond to differences in post-thymic maturation, it is important to directly compare RTEs from neonatal and adult mice. If neonatal CD8^+^ T cells behave differently from adult CD8^+^ T cells because they comprise more RTEs, then age-related differences in CD8^+^ T cells should disappear when we directly compare neonatal and adult RTEs. Unfortunately, comparing neonatal and adult CD8^+^ RTEs is challenging because there is currently no marker to distinguish RTEs from mature CD8^+^ T cells, and the experimental strategies that have been developed to label RTEs in mice have well-known limitations. For example, injection of fluorescein isothiocyanate (FITC) into the thymus can mark a small number of thymocytes that later become FITC^+^ RTEs in the periphery, but the introduction of surgical stress may alter the behavior of RTEs.^20^^,^^21^ Others have used bromodeoxyuridine (BrdU) incorporation to label the rapidly dividing thymocytes before these thymocytes are exported into the periphery as RTEs, but BrdU can also be incorporated into mature T cells that are undergoing cell division.^22^^,^^23^ Perhaps, the most useful method for identifying RTEs is to use Rag2p-GFP transgenic (Tg) mice, whereby GFP expression remains detectable in peripheral T cells for a finite period of time (∼3 weeks), thereby serving as a marker for RTEs.^4^^,^^5^^,^^24^ However, the major disadvantage of this approach is that the GFP signal is lost in cells that undergo extensive homeostatic proliferation, which makes them unsuitable for identifying RTEs in the more lymphopenic neonatal mice.^25^^,^^26^

In this study, we used a new method to identify RTEs in both neonatal and adult mice. Our approach involved the use of fate-mapping mice to permanently label, or “timestamp,” a wave of thymic CD8^+^ T cells at the time of tamoxifen exposure.^14^^,^^27^^,^^28^ The advantage of this approach is that we can identify neonatal and adult RTEs in the absence of surgical stress, and the label is not diluted out by proliferation. Along with timestamping the mice, we employed the 10× Genomics Chromium Single Cell Immune Profiling platform (paired T cell receptor [TCR]/RNA-seq) to rigorously compare how the composition of RTEs differs between neonatal and adult mice and performed flow cytometry to assess their phenotype and functions. These experiments demonstrated that all CD8^+^ RTEs are not created equally and that the altered proportion of RTEs in neonatal and adult animals is not a major contributor to the age-related differences in CD8^+^ T cell behavior.

## Results

### Phenotypes of neonatal and adult CD8^+^ RTEs

**Figure 1:**
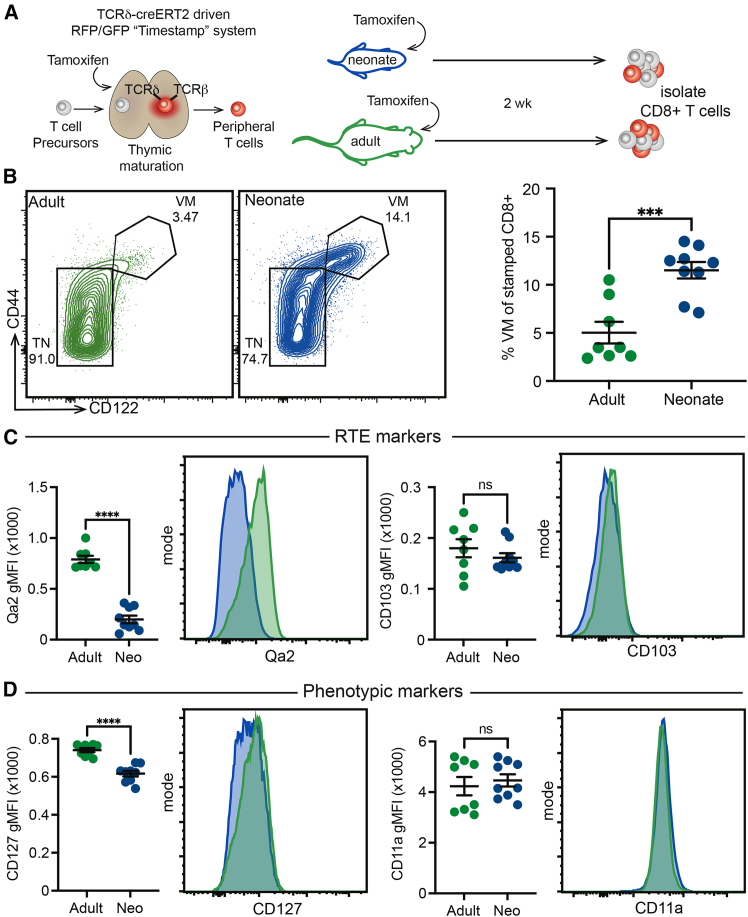
Neonatal and adult RTEs are phenotypically distinct (A) Schematic of the timestamping system marking T cells made at the time of tamoxifen administration. RTEs were collected at 2 weeks post-marking. (B) Left: Representative contour plots displaying virtual memory (VM, CD44^hi^ CD122^hi^) and true naive (TN, CD44^lo^ CD122^lo^) CD8^+^ populations. Right: Statistical analysis of VM population within the marked T cell population. (C) Statistical analysis and representative histograms of the RTE markers Qa2 (left) and CD103 (right). (D) Statistical analysis and representative histograms of the phenotypic markers CD127 (left) and CD11a (right). n = 8-9 mice per group from 2 independent experiments. For statistical analysis, unpaired *t* tests were performed. ns, not significant; ∗∗∗, *p* < 0.001; ∗∗∗∗, *p* < 0.0001.

The goal of this study was to determine whether CD8^+^ RTEs in neonatal and adult mice are phenotypically and functionally distinct. To accomplish this goal, we used a fate-mapping mouse model that allowed us to permanently label RTEs produced at different ages. This model is based on the TCRδ-CreER strain.^29^^,^^30^ The TCRδ constant gene is expressed in all T cells in the thymus at the double-negative (DN) and double-positive (DP) stages of development but is excised in αβ T cells during their transition to the single-positive (SP) stage of thymopoiesis.^31^^,^^32^ Thus, by crossing the TCRδ-creERT2 mice with a reporter strain that has a *loxP*-flanked “stop” cassette upstream of a fluorescent reporter in the Rosa26 locus (R26R^ZsGreen^ or R26R^TdTomato^), we excised the “stop” cassette and permanently labeled, or “timestamped,” a wave of CD8^+^ T cells made in the thymus only during the time of tamoxifen exposure. Although other T cell fate-mappers (e.g., CD4-creERT2 mice^33^) could be used to label RTEs, we elected to use TCRδ-creERT2 mice because they have low background marking (in the absence of tamoxifen), allow for the labeling of other types of T cells (not examined in this study), and have a precise window of timestamping (Figure S1A^30^). To start, we administered tamoxifen to one group of TCRδ-creERT2 mice at 1 day of age to mark a wave of neonatal CD8^+^ T cells and another group of TCRδ-creERT2 mice at 28 days of age to label a wave of adult CD8^+^ T cells (see STAR Methods) (Figure 1A). Two weeks after labeling, we collected timestamped CD8^+^ RTEs from the spleens of both groups of mice and compared their phenotype by using flow cytometry (Figure S2A).

We previously reported that a large proportion of CD8^+^ T cells in neonatal mice exhibit a virtual memory (VM) phenotype (CD44^hi^CD122^hi^), whereas only a small fraction of CD8^+^ T cells in adult mice show this phenotype.^10^^,^^34^ Thus, we were interested in determining whether this phenotypic difference would still be evident in neonatal and adult CD8^+^ RTEs that had undergone the same amount of post-thymic maturation. Interestingly, the neonatal CD8^+^ RTEs contained significantly more VM cells than their adult counterparts (Figure 1B). The neonatal and adult RTEs expressed different amounts of the RTE maturation marker Qa2 but similar levels of CD103 (Figure 1C). Neonatal RTEs also had lower expression of the IL-7 receptor CD127 but similar levels of CD11a (Figure 1D). Thus, many surface markers that have traditionally been used to identify immature naive T cells do not apply to CD8^+^ T cells produced in early life.

We considered the possibility that some of the phenotypic differences between neonatal and adult RTEs were driven by changes in the peripheral environment (e.g., availability of homeostatic cytokines). In particular, neonatal CD8 RTEs are exported into a more lymphopenic environment, which may promote homeostatic proliferation and accumulation of VM cells.^25^^,^^26^ To control for age-related differences in the peripheral environment, we used a dual-reporter timestamp system in which a thymic lobe from a 1-day-old TdTomato^+^ timestamped animal is transplanted under the kidney capsule of a 6-week-old ZsGreen^+^ timestamped animal (Figure S1B). In this way, we could compare the phenotypes of neonatal and adult CD8^+^ RTEs in the same peripheral environment. Consistent with our findings in different-aged mice, we found that neonatal CD8^+^ RTEs acquire a unique phenotype, even when matured in a lymphoreplete adult environment (Figures S1C and S1D). Furthermore, we found that neonatal RTEs show higher expression of the transcription factor Eomes (Figure S1E) and retain elevated levels of Ki-67 (Figure S1F) after that are exported into the adult peripheral environment. Collectively, these results suggest that phenotypic differences between bulk neonatal and adult CD8^+^ T cells cannot be solely attributed to differences in the amounts of post-thymic maturation.

### Gene expression profiles of neonatal and adult CD8^+^ RTEs

We previously found that neonatal and adult CD8^+^ T cells exhibited a distinct gene expression profile, even before they were exported from the thymus.^10^^,^^35^ However, these studies were performed at the bulk population level and did not compare mature CD8^+^ T cells from neonatal and adult mice that had undergone similar amounts of maturation. To understand how the composition and function of the RTE pool changes at different stages of life, we examined the single-cell gene expression profiles of RTEs produced within neonatal and adult mice. We employed paired single-cell RNA sequencing (scRNA-seq) and single-cell TCR sequencing (scTCR-seq) to obtain both gene expression and TCR profiles from individual CD8^+^ T cells “stamped” in neonatal and adult mice. The stamped CD8^+^ T cells were sorted from the spleens of neonatal and adult mice at two weeks post stamping (Figure S2B), which allowed us to examine gene expression profiles in the same two groups of RTEs that were analyzed by flow cytometry.

**Figure 2:**
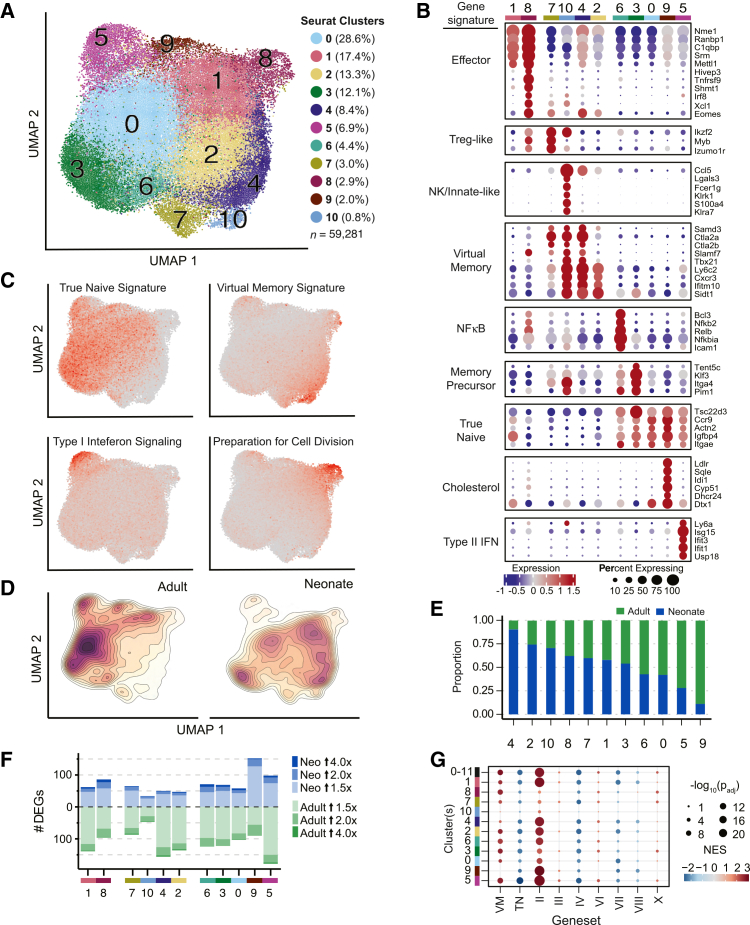
RTE pools possess age-related differences in the composition and gene expression (A) UMAP visualization of neonate (32,185) and adult (27,096) RTE transcriptomes. Cluster identities are indicated by color, and cluster size is conveyed as a percentage of the total number of RTEs sequenced (59,281). *N* = 4 mice for both neonatal and adult groups. (B) Bubble plot depicting the expression of the top 5 marker genes. Cluster-specific genes based on their average expression values across the clusters. Gene signature labels provide cluster annotations. Dot shading indicates the mean normalized expression (row-wise *Z* score), and dot sizes represent the percentage of cells expressing each gene. (C) UMAP visualization of defined gene set module scores. Single-cell gene set module scores are conveyed by point color. (D) Density estimation of single cells within the UMAP projection, split by age. Filled contour lines indicate regions of the UMAP with high and low densities of cells. (E) Proportion of adult- and neonatal-derived RTEs that constitute each cluster, normalized to the total number of adult and neonatal RTEs profiled. Clusters ordered from the highest to the lowest proportion of neonatal cells. (F) Number of age-related differentially expressed genes per cluster. Fill color indicates the fold-change magnitude; blue fill shades indicate DEGs upregulated in neonatal RTEs, and green fill shades indicate DEGs upregulated in adult RTEs. All counted DEGs have a base mean expression value greater than 20. (G) Bubble plot depicting the GSEA results when comparing the per cluster age-related differential expression results to the virtual memory (VM), true naive (TN), and ImmGen T cell gene signature (II, III, IV, VI, VII, VIII, and X) gene sets. Dot shading indicates the magnitude and direction of the NES, while dot sizes indicate the adjusted *p* value, with larger dots corresponding to smaller adjusted *p* values. The “0–10” row displays the enrichment results calculated by comparing all neonatal RTE transcriptomes with all adult RTE transcriptomes. UMAP, uniform manifold approximation and projection; GSEA, gene set enrichment analysis; NES, normalized enrichment score.

To characterize the heterogeneity of the RTE pool, we performed dimensionality reduction and clustering of the 59,281 single-cell transcriptome profiles, using Seurat^36^ (Figure 2A). We identified a total of 11 clusters and assigned functional annotations to each cluster, considering both the cluster-specific upregulation of individual genes (Figure 2B) and gene set module scores, a method that reports the expression of multiple genes within a pathway of interest at single-cell resolution^37^ (Figure 2C). Broadly, the 11 clusters partitioned into three functionally distinct superclusters: (1) effector-like, (2) true naive-like, and (3) VM-like superclusters. Clusters 1 and 8 made up the effector-like supercluster, expressing genes typically upregulated post activation (Figure 2B; *Nme1*, *Srm*, and *Shmt1*) and having transcriptomes with high gene set module scores for “preparation for cell division” (Figure 2C). Clusters 0, 3, 5, 6, and 9 made up the true naive-like supercluster, expressing genes associated with a naive state (Figure 2B; *Tsc22d3* and *Ccr9*)^38^^,^^39^ and possessing transcriptomes with the highest “true naive” gene set module scores (Figure 2C). Conversely, clusters 2, 4, 7, and 10 made up the VM-like supercluster, expressing genes associated with a VM phenotype (Figure 2B; *Smad3*, *Tbx21*, *Ly6c2*, and *Cxcr3*)^14^ and having transcriptomes with the highest “VM” gene set module scores (Figure 2C).

To identify the transcriptional programs of individual clusters within each supercluster, we performed gene set enrichment analysis (GSEA),^40^ querying genes distinctly upregulated or downregulated within each cluster against ImmGen, KEGG, and ImmPort curated pathways.^41^^,^^42^^,^^43^ Within the effector-like supercluster (clusters 1 and 8), cluster 8 transcriptomes possessed particularly high enrichment for gene sets associated with an activated state, including “lymphocyte activation,” “initial cytokine or effector response (I),” and “glycine, serine, and threonine metabolism” (Figures S3A–S3C). Within the true naive-like supercluster (clusters 0, 3, 5, 6 and 9), cluster 3 transcriptomes were distinctly enriched for the “memory precursor (VII)” gene signature; notably, enrichments were driven by the upregulation of the memory-associated genes *Il7r, Tcf7*, and *Bcl2*^43^ (Figure S3B). Interestingly, despite their overall true naive transcriptional phenotype, clusters 5, 6, and 9 expressed genes signifying a response to external stimuli. Cluster 5 transcriptomes were enriched for the “interferon alpha/beta signaling” gene signature, corresponding to the recently defined ISAG^hi^ (interferon signaling-associated gene) subset^44^^,^^45^ (Figure S3C). Cluster 6 transcriptomes were uniquely enriched for NF-κB-dependent pathways, such as “toll-like receptor cascades” and “T cell activation,” possessing an upregulation of NF-κB subunits (*Nfkb2* and *Relb*) and targets (*Bcl3* and *Icam1*) (Figure S3C). Cluster 9 transcriptomes were enriched for “steroid biosynthesis,” potentially indicating an effector-primed metabolism, given the upregulation of cholesterol production following T cell activation^46^ (Figure S3A). Within the VM supercluster (clusters 2, 4, 7, and 10), cluster 7 uniquely expressed high levels of the Treg-associated transcripts *Izumo1r*, *Ikzf2*, and *Ctla4* (Figure 2B). Finally, while clusters 4 and 10 shared enrichment for the “short-term effector and memory (VI)” gene signature, cluster 10 transcriptomes shared gene expression features with subsets of innate-like lymphocytes, highlighted by the distinct enrichment of gene sets associated with NK cell function and activation of an innate immune response (Figures S3B and S3C).

Having characterized the phenotypic composition of the RTE pool, we next asked how the neonatal and adult RTE pools differed, first hypothesizing that the RTE pools could differ because of age-related differences in the proportion of cells from each of the defined clusters. To address this possibility, within each cluster, we compared the proportion of cells derived from neonates with the proportion of cells derived from adults. We found that neonatal RTEs contributed more to the VM clusters 2 and 4, whereas adult RTEs contributed more to the true naive-like clusters 5 and 9 (Figures 2D and 2E). Therefore, neonatal and adult RTE pools may be biased toward different infection responses due to differences in their phenotypic composition in the steady state.

In addition to differences in the cluster composition of the RTE pool, age-related differences in gene expression within each cluster could also impact how the neonatal and adult RTE pools respond to infection. Indeed, neonatal and adult RTE pools also possessed core transcriptomic differences; by performing a differential expression analysis to compare neonatal and adult transcriptomes on a per-cluster basis, we identified 897 differentially expressed genes (Figure 2F). To determine whether the age-related differences in gene expression were either consistent across the defined clusters or cluster specific, we first employed a *k*-means clustering approach, which revealed 6 clusters of genes differentially expressed by age. Within each of the identified *k*-means clusters, the differentially expressed genes were either upregulated across nearly all phenotypic clusters in neonatal RTEs (K1, K2, and K6) or upregulated across nearly all phenotypic clusters in adult RTEs (K3, K4, and K5) (Figure S3D). To gain insight into the functional significance of the core transcriptomic differences between neonatal and adult RTEs, GSEA was performed using the per-cluster, age-related differential expression results. The analysis revealed positive enrichments (indicating upregulation in neonatal RTEs) for the “VM,” “preparation for cell division (II),” “late effector or memory (X),” “short-term effector and memory (VI),” and “cell cycle and division (III)” gene signatures. Conversely, the “true naive,” “naive and late memory (IV),” “memory precursor (VII),” and “naive or late effector or memory (VIII)” gene signatures were negatively enriched, indicating upregulation of their constituent genes in adult RTEs (Figure 2G). The consistency of the enrichment results across the phenotypic clusters demonstrated that all neonatal RTEs in the neonatal RTE pool possess not only more cells with a VM phenotype but also, regardless of their phenotype (e.g., true naive and VM), a more effector-biased transcriptome compared with adult RTEs.

### TCR usage in neonatal and adult CD8^+^ RTEs

We next sought to determine the key factors that contribute to the altered gene expression profiles between neonatal and adult RTEs. For example, why do neonatal CD8^+^ RTEs exhibit a more VM phenotype? One possibility is that the altered developmental trajectory among neonatal RTEs corresponds to their usage of different TCRs. To examine this possibility, we analyzed the TCR data in our paired scTCR/RNA-seq datasets for neonatal and adult RTEs. We first asked whether the TCR composition was skewed in CD8^+^ RTEs produced in early life. When we compared V gene usage, we observed a similar pattern of V gene expression in both the TCRβ and TCRα chains of RTEs produced in neonatal and adult mice (Figures S4A and S4B). We did not observe a significant difference in J gene usage in neonatal and adult CD8^+^ RTEs, another similarity between the two groups (Figures S4C and S4D). Thus, despite their distinct phenotypes, the usage of V and J genes was largely comparable between both neonatal and adult CD8^+^ RTEs.

For a more in-depth comparison, we then focused on features in the CDR3 regions of the TCRβ and TCRα chains. Previous work has demonstrated that neonatal T cells have more germline-encoded TCRs (with no N-nucleotide addition) than adult T cells, as the expression of TdT (the enzyme responsible for the insertion of N-nucleotides in the junctional regions) is absent in the thymus of mice until 4–8 days of age.^47^^,^^48^ Consistent with these studies, we found that the timestamped CD8^+^ T cells in neonatal mice showed a significantly higher proportion of cells with no N-nucleotide addition in the TCRβ chain compared with their counterparts in adult mice (Figure S4E). The lack of junctional diversity in neonatal CD8^+^ RTEs also translated into shorter CDR3β lengths. In contrast, we did not observe significant age-related differences in the numbers of N-nucleotide additions to the TCRα chain (Figure S4F), which is consistent with the findings of earlier work.^49^ We also found a smaller number of unique clonotypes in neonatal CD8^+^ RTEs (Figure S4G), suggesting that the altered phenotype of CD8^+^ T cells made in early life corresponds to a less diverse pool of T cells, which is biased toward shorter and more germline-encoded TCRs.

**Figure 3:**
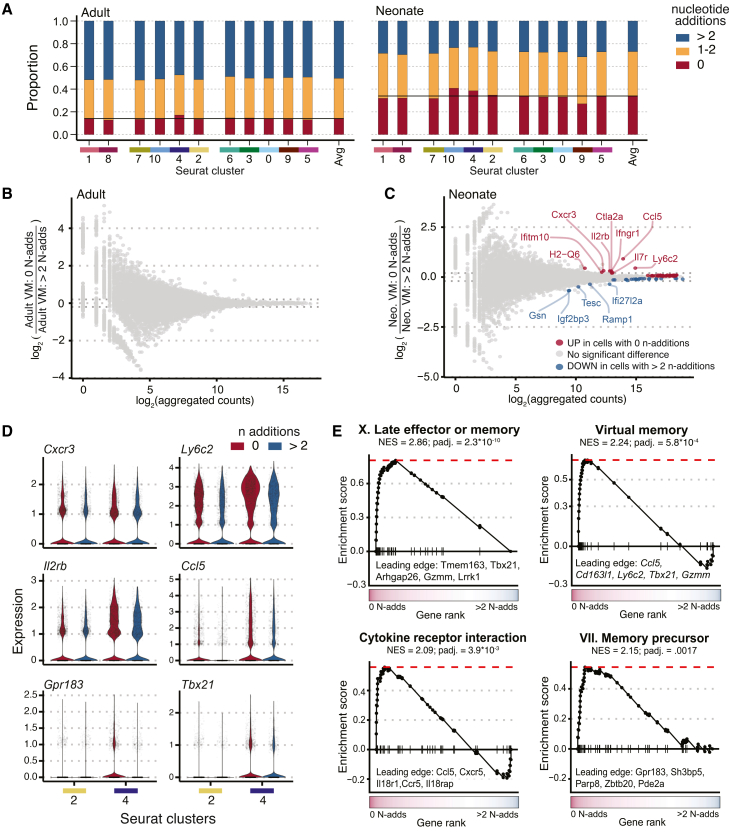
TCR complexity is associated with the effector-biased transcriptome of neonatal RTEs (A) Proportion of RTEs with different numbers of TCRβ N-nucleotide additions. Proportions were calculated after the cells were grouped by gene expression clusters and age. Fill color indicates the number of N-nucleotide additions, binned into 0, 1–2, and >2 groups. (B and C) MA plots visualizing the relationship between gene expression (*x* axis) and log_2_ (fold-change) when comparing either adult RTEs (B) or neonatal RTEs (C) that possess a VM transcriptome (gene expression clusters 2 and 4) and TCRβs with 0 N-nucleotide addition versus RTEs that possess a VM transcriptome and TCRβs with >2 N-nucleotide additions. Each point represents a gene, and point color represents the significance and direction of the differential expression result. All red and blue points have an adjusted *p* value of <0.05, while labeled points have a log_2_ (fold-change), with an absolute value of >0.20. (D) Violin plots showing the distribution of single-cell gene expression (*y* axis) for *Cxcr3*, *Ly6c2*, *Il2rb*, *Ccl5*, *Gpr183*, and *Tbx21*. Single-cell expression values are grouped along the *x* axis by the gene expression cluster (columns labeled 2 and 4) and the number of N-nucleotide additions (red = “0” and blue = “>2”). *Gpr183* and *Tbx21* are not statistically significantly differentially expressed, but they appear within the leading edge of enriched pathways in (E). (E) GSEA running enrichment score plots for four selected pathways significantly upregulated in neonatal VM phenotype RTEs with 0 N-nucleotide addition TCRβs compared with >2 N-nucleotide addition TCRβs (gene rank is expressed as mean log2 [*Neo*. VM: 0 N-nucleotide adds/*Neo*. VM: >2 N-nucleotide adds]).

Finally, we sought to determine whether the bias toward VM cells is linked to the usage of more germline-encoded TCRs. To address this question, we compared the proportion of cells with germline-encoded TCRs across each gene expression cluster and found that the germline-encoded RTEs were slightly more prevalent in clusters 4 (neonatal and adult) and 10 (neonate only), both of which possess a VM phenotype (Figure 3A). Further, to understand whether neonatal RTEs that express TCRs with zero N-nucleotide addition possess a heightened effector-like phenotype, we performed differential expression analysis, comparing VM RTEs with zero N-nucleotide addition to neonatal VM RTEs with >2 N-nucleotide additions (Figures 3B and 3C) and found that neonatal VM cells with germline-encoded TCRs upregulated genes (*Cxcr3*, *Ly6c2*, and *Il2rb*) that are associated with the VM phenotype (Figure 3B). GSEA revealed that the “late effector or memory (X),” “VM,” “cytokine-cytokine receptor interaction,” and “memory precursor (VII)” gene signatures were positively enriched, indicating a coordinated upregulation of T cell response-associated genes (*Ccl5*, *Gpr183*, and *Tbx21*) within neonatal cells possessing germline-encoded TCRs (Figures 3D and 3E). Although these differences may be driven by a small effect size, we did not observe differentially expressed genes or strong GSEA results when comparing the gene expression profiles of adult RTEs with zero N-nucleotide addition versus those with >2 N-nucleotide additions (Figures 3B and S4H), indicating that germline-encoded TCRs exhibit a distinct transcriptional signature only in early life. Collectively, these data suggest that neonatal CD8^+^ RTEs with the most effector-like gene expression profiles tend to express germline-encoded TCRs.

### Functions of neonatal and adult RTEs

**Figure 4:**
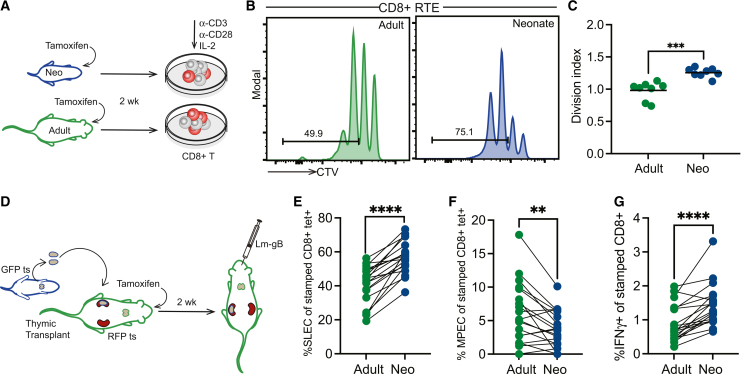
Neonatal RTEs exhibit enhanced immune functionality (A) Schematic of *in vitro* stimulation. CD8^+^ T cells from neonatal and adult timestamped (ts) mice were coated with CTV and stimulated for 48 h via antibody-mediated crosslinking of CD3/CD28. (B) Representative histograms of CTV dilution. (C) Division index. Unpaired *t* test was performed for statistical analysis. n = 8 samples per group from 2 independent experiments. (D) Schematic of the *in vivo* infection experiment. Thymic lobes from newborn GFP timestamped mice were grafted under the kidney capsule of adult RFP timestamped mice. Tamoxifen was administered to thymic graft recipients, and after 2 weeks, recipients were infected with 5 × 10^3^ CFUs of LM-gB. Spleens were collected at 5 dpi. (E) Percentage of timestamped tetramer-positive cells with a short-lived effector phenotype (KLRG1^hi^CD127^lo^). (F) Percentage of timestamped tetramer-positive cells with a memory precursor effector phenotype (KLRG1^lo^CD127^hi^). (G) Percentage of timestamped cells producing IFN-γ. *N* = 19. For statistical analysis, paired *t* tests were performed. ∗∗, *p* < 0.01; ∗∗∗, *p* < 0.001; ∗∗∗∗, *p* < 0.0001.

An important question is whether neonatal and adult RTEs exhibit distinct functional properties. We previously showed that neonatal CD8^+^ T cells undergo more cell divisions than adult CD8^+^ T cells after *in vitro* TCR stimulation and preferentially give rise to short-lived effectors (KLRG1^+^ CD127) after *in vivo* infection.^9^^,^^10^^,^^11^^,^^50^ However, these experiments were performed with bulk CD8^+^ T cells from neonatal and adult animals, raising the possibility that the increased percentage of RTEs in the neonatal pool may be underlying these age-related differences. To examine this possibility, we again used our timestamp mice to generate neonatal and adult CD8^+^ RTEs. This time, we coated the neonatal and adult RTEs with a proliferation dye (CellTrace Violet, CTV) and compared their ability to proliferate after *in vitro* stimulation with plate-bound anti-CD3/anti-CD28 (Figure 4A). Two days after stimulation, we examined the dilution of CTV, a proliferation metric, and found that neonatal RTEs had undergone more divisions than adult RTEs, which could relate to having increased numbers of VM cells in the starting pool (Figures 4B and 4C). Thus, even after controlling for age-related differences in post-thymic maturation, neonatal CD8^+^ T cells were more proliferative than adult CD8^+^ T cells.

To control for age-related differences in the peripheral environment, we repeated our thymic transplant experiments. For these studies, we administered tamoxifen after thymic transplantation to generate waves of neonatal (ZsGreen) and adult (TdTomato) CD8^+^ RTEs in the same host (Figure S5A). Importantly, neonatal CD8^+^ RTEs still underwent more proliferation than adult RTEs after *in vitro* TCR stimulation (Figures S5B and S5C). We also infected the dual timestamped thymic transplant mice with a recombinant strain of *Listeria monocytogenes* that expresses the gB peptide from HSV-1 (denoted LM-gB; Figure 4D) and compared the ability of neonatal and adult RTEs to differentiate into effector cells (see gating strategy, Figure S2C, for identification of antigen-specific RTEs). Strikingly, a higher proportion of neonatal RTEs displayed a short-lived effector phenotype (SLEC; Figure 4E) and fewer memory precursor effector cells (MPECs; Figure 4F). The neonatal RTEs also produced more effector molecules such as IFN-γ (Figure 4G), granzyme A, granzyme B, and TNF-α (Figures S5D–S5F) than adult RTEs after peptide stimulation. In contrast, the adult RTEs generated fewer SLECs and more MPECs. Collectively, these data suggest that post-thymic maturation contributes very little to the differences between neonatal and adult CD8^+^ T cells; instead, such differences are largely cell intrinsic.

## Discussion

In this report, we leveraged a new tool to identify RTEs in neonatal and adult animals, which allowed us to directly compare their phenotype and functions. We found that neonatal CD8^+^ RTEs are distinguishable from adult CD8^+^ RTEs by their expression of distinct surface markers, gene expression profiles, and TCR features. The neonatal CD8^+^ RTEs also responded more rapidly to stimulation than adult CD8^+^ RTEs and gave rise to more terminally differentiated effector CD8^+^ T cells after infection. These data indicate that the RTE phenotype is dependent on the age of the animal and that post-thymic maturation is not a major driver of cell-intrinsic differences between neonatal and adult CD8^+^ T cells.

This study builds upon our earlier work showing how CD8^+^ T cells made in early life are phenotypically and functionally distinct from those produced in adulthood.^9^^,^^11^^,^^51^ Although we previously showed that many of the age-related differences in CD8^+^ T cells stem from their distinct developmental origins,^10^^,^^14^ there is still a significant amount of heterogeneity in the neonatal pool for reasons that are not clear. In this report, we examined how other key factors (post-thymic maturation and TCR usage) contribute to phenotypic and functional variations within the CD8^+^ T cell pool. We again used timestamp mice, but instead of looking at the role of neonatal CD8^+^ T cells in adult mice, we used timestamped mice to control for age-related differences in post-thymic maturation; so, we could determine how TCR usage contributes to heterogeneity within the neonatal and adult pools. The timestamp approach is necessary for these studies because the adult pool of CD8^+^ T cells contains neonatal cells, and the phenotype of cells changes with the amount of post-thymic maturation. Consistent with the results of previous studies, we found that neonatal CD8^+^ T cells exhibit shorter and more germline-encoded TCRs.^52^^,^^53^ However, by simultaneously examining gene expression and TCR sequences in individual cells, we discovered that germline-encoded TCRs exhibit a bias toward becoming VM cells in early life.

The association between germline-encoded TCRs and VM phenotype in neonatal CD8^+^ RTEs is reminiscent to the thymic programming of γδ T cells possessing germline-encoded TCRs in the thymus in early life.^54^^,^^55^ Although it is not clear why neonatal RTEs with germline-encoded TCRs have a propensity to become VM cells, several possibilities are worth mentioning. First, germline-encoded TCRs have been shown to be more cross-reactive, or “peptide-promiscuous,”^56^ which may enable them to receive stronger signaling in response to self-peptide:MHC complexes in the periphery. Second, TCRs with more N-nucleotide additions tend to be longer, and longer TCRs may impair MHC binding and TCR signaling in the thymus.^57^ Third, the germline-encoded TCRs may correspond to an early wave of hematopoietic progenitors that are programmed differently. Indeed, recent studies have shown that the thymus is colonized by multiple waves of hematopoietic progenitors.^58^^,^^59^^,^^60^ However, the TCR repertoire of CD8^+^ T cells derived from each wave has yet to be explored.

Previous studies have uncovered distinct transcriptional states in the naive CD4 pool, including some clusters that are distinguished by their expression of TCR pathway genes, memory-like genes, or interferon-response genes.^61^^,^^62^ Interestingly, we observed similar clusters in the CD8^+^ RTE pool, which are preferentially populated by cells made at different stages of life. The CD8^+^ T cells produced in neonatal mice have a propensity to express more memory-like genes, whereas those made in adult mice tend to give rise to the subsets that are enriched for interferon responsiveness and cholesterol biosynthesis. By using timestamped mice, we were able to compare CD8^+^ T cells that were definitively “neonatal” or “adult” and demonstrate how the time of production contributes to transcriptional heterogeneity in the naive T cell pool.

In the future, it will be important to examine whether similar age-related differences exist in human CD8^+^ T cell RTEs. It would also be useful to know which neonatal cell subsets and TCRs are preferentially preserved in the adult T cell pool. Indeed, we previously demonstrated that some (but not all) neonatal CD8^+^ T cells persist into adulthood, and the ones that persist are the first CD8^+^ T cells to respond to infection.^14^ However, the transcriptional and clonotypic features of these long-term survivors have remained a mystery. Similarly, we do not yet know which transcriptional and TCR features are present on the neonatal cells that preferentially give rise to short-lived effectors in adult animals. While these questions remain to be answered, this study provides new insight into how CD8^+^ T cells are made differently at various stages of life.

### Limitations of the study

The main limitation of the study is that our conclusions are based on a specific set of experimental conditions in mice. For example, we provided tamoxifen to timestamped mice at birth and 28 days of age to label waves of neonatal and adult CD8^+^ T cells. However, it is possible that the phenotypes and function of CD8^+^ T cells produced in early life and adulthood depend on the precise time at which tamoxifen is administered. Also, we compared the functions of neonatal and adult CD8^+^ T cells at 2 weeks post-marking because this is the time when labeled CD8^+^ T cells are first detected in the periphery. However, post-thymic maturation in mice takes 3 weeks, and it is possible that age-related differences in RTEs depends on the precise time (during the maturation period) at which neonatal and adult RTEs are compared. Lastly, we compared the ability of neonatal and adult RTEs to respond to LM-gB, but further studies are required to determine how generalizable our conclusions are to other types of infections and epitope-specific CD8^+^ T cells.

## Resource availability

### Lead contact

All requests for further information or resources generated in this study should be directed to and will be fulfilled by lead contact, Brian Rudd (bdr54@cornell.edu).

### Materials availability

This study did not generate new materials or unique reagents.

### Data and code availability

• Data: Flow cytometry files will be available upon request to the lead contact. Sequencing data in this paper will be made freely available upon publication and can be found via accession number GEO: GSE288766.
• Code: No original code was generated in this study.
• Other items: Any other items or information needed to reanalyze the data will be made available upon request.

## Acknowledgments

We thank the Flow Cytometry Facility (RRID: SCR_021740) for expert sorting assistance and the BRC Genomics Facility (RRID: SCR_021727) for the generation and sequencing of libraries. This work was supported by 10.13039/100000002National Institutes of Health awards R01HD107798, R01AI05265, R01AI110613, and R01AI189855 (to B.D.R from the National Institute of Child Health and Human Development and 10.13039/100000060National Institute of Allergy and Infectious Diseases), R21AI138025 (to N.L.S. from 10.13039/100000060National Institute of Allergy and Infectious Diseases), and F31AI157236 (to C.T. from 10.13039/100000060National Institute of Allergy and Infectious Diseases), as well as the Australian National Health and Medical Research Council Career Development Fellowship 1067590 (to V.V.).

## Author contributions

Conceptualization, M.P.D. and B.D.R.; data curation, N.L.S.; formal analysis, C.T., V.V., C.K., J.K.G., and N.L.S.; investigation, C.T., V.V., C.K., J.K.G., M.P.D., and N.L.S.; writing – original draft, C.T., V.V., C.K., N.L.S., and B.D.R.; writing – review & editing, A.M., P.G.T., A.G., and M.P.D.; resources, A.M., P.G.T., and A.G.; funding acquisition, A.G., M.P.D., and B.D.R.; supervision, A.G.; project administration, N.L.S. and B.D.R.

## Declaration of interests

The authors declare no competing interests.

## STAR★Methods

### Key resources table

**Table:** 

REAGENT or RESOURCE	SOURCE	IDENTIFIER
**Antibodies**		

Anti-mouse CD3 (clone 2C11)	Invitrogen	Cat# 16-0031-85; RRID:AB_468848
Anti-mouse CD28 (clone 37.51)	Invitrogen	Cat# 16-0281-85; RRID:AB_468922
Anti-mouse CD4 (clone GK1.5)	BD Biosciences	Cat# 612900; RRID:AB_2827960
Anti-mouse CD8 (clone 53-6.7)	Biolegend	Cat# 104011; RRID:AB_3097648
Anti-mouse CD44 (clone IM7)	BD Biosciences	Cat# 612799; RRID:AB_2870126
Anti-mouse CD122 (clone TM-β1)	BD Biosciences	Cat# 562960; RRID:AB_2737918
Anti-mouse Qa2 (clone 1-1-2)	BD Biosciences	Cat# 743309; RRID:AB_2871499
Anti-mouse CD103 (clone 2E7)	Biolegend	Cat# 121416; RRID:AB_2128621
Anti-mouse CD127 (clone A7R34)	Invitrogen	Cat# 12-1271-83; RRID:AB_465845
Anti-mouse CD11a (clone M17/4)	BD Biosciences	Cat# 740866; RRID:AB_2740518
Anti-mouse KLRG1 (clone 2F1)	BD Biosciences	Cat# 562897; RRID:AB_2737875
Anti-mouse IFNγ (clone XMG1.2)	BD Biosciences	Cat# 563773; RRID:AB_2738419
Anti-mouse TNFα (clone MP6-XT22)	BD Biosciences	Cat# 563943; RRID:AB_2738498
Anti-mouse Granzyme A (clone GzA-3G8.5)	Invitrogen	Cat# 46-5831-82; RRID:AB_2573775
Anti-human/mouse Granzyme B (clone GB11)	BD Biosciences	Cat# 563389; RRID:AB_2738175
Anti-mouse Ki67 (clone SolA15)	Invitrogen	Cat# 47-5698-82; RRID:AB_2688065
Anti-mouse Eomes (clone Dan11mag)	Invitrogen	Cat# 61-4875-82; RRID:AB_2574614

**Bacterial and virus strains**		

*Listeria monocytogenes*; expressing HSV-1 gB peptide	Orr et al. 2007^64^	N/A

**Chemicals, peptides, and recombinant proteins**		

Tamoxifen	Sigma	Cat# T5648
recombinant human IL-2	Thermo Fisher Scientific	Cat# 14-8029-81
RPMI	Corning	Cat# 10-040-CV
Fetal bovine serum	VWR/Seradigm	Cat# 97068-085
Penicillin/Streptomycin	Cytiva	Cat# SV30010
L-glutamine	Invitrogen	Cat# 25030-081
Biotinylated gB monomer	NIH Tetramer core	H2-Kb | HSV-1 gB 498–505 | SSIEFARL
APC streptavidin	Invitrogen	Cat# S-868
gB peptide: SSIEFARL	21^st^ Century biochemical	Custom synthesis
eBiosciences Fixable Viability Dye eFluor 780	Invitrogen	Cat# 65-0865-18

**Critical commercial assays**		

Chromium Single Cell Universal V(D)J Reagent kits (V1.1 Chemistry)	10x Genomics	Cat# PN-1000167; PN-1000020; PN-1000071; PN-1000127; PN-10000213
Cell trace violet	Invitrogen	Cat# 2776002
IC fix buffer set	Thermo Fisher Scientific	Cat# 88-8824-00
BD fix/perm buffer set	BD Biosciences	Cat# 554714
BD perm plus	BD Biosciences	Cat# 651651
CD8a Microbeads, mouse	Miltenyi Biotec	Cat# 130-117-044

**Deposited data**		

Raw and analyzed data	This paper	GSE288766

**Experimental models: Organisms/strains**		

Mouse: B6.Cg-Gt(ROSA)26Sor^tm6(CAG−ZsGreen1)Hze^/J	The Jackson Laboratory	RRID: IMSR_JAX:007906
Mouse: B6.Cg-Gt(ROSA)26Sor^tm9(CAG-tdTomato)Hze^/J	The Jackson Laboratory	RRID: IMSR_JAX:007909
Mouse: B6.129S-Tcrd^tm1.1(cre/ERT2)Zhu^/J	The Jackson Laboratory	RRID: IMSR_JAX:031679

**Software and algorithms**		

Flowjo	BD Biosciences	RRID:SCR_008520
FACSdiva	BD Biosciences	RRID:SCR_001456
Prism	GraphPad	RRID:SCR_002798
Cell Ranger v6.0.0	10x genomics	RRID:SCR_023221
R version v4.3.2	R Core Team^70^	RRID:SCR_001905
Seurat v5.0.0	Hao et al. 2024^36^	RRID:SCR_016341
ggplot2 v3.5.2	Wickman. 2016^67^	RRID:SCR_014601
scCustomize v2.1.2.9029	Marsh. 2025^65^	RRID:SCR_024675
UCell v2.6.2	Andreatta. 2021^37^	RRID:SCR_027109
fgsea v1.28.0	Korotkevich et al. 2021^66^	RRID:SCR_020938
Matrix.Utils v0.9.7	Varrichio. 2024^68^	https://github.com/cvarrichio/Matrix.utils
stats v4.3.2	R Core Team^70^	RRID:SCR_025968
factoextra v1.0.7	Kassambara 2020^71^	RRID:SCR_016692
DESeq2 v1.42.1	Love et al. 2014^69^	RRID:SCR_015687
IMGT/HighV-QUEST	Alamyar et al. 2012^72^	RRID:SCR_018196

**Other**		

BRC Genomics Core	Cornell University	RRID:SCR_021727
BRC Flow Cytometry Core	Cornell University	RRID:SCR_021740

### Experimental model and study participant details

The experimental model used for this study is a murine fate mapping model designed to track CD8^+^ T cells made at different developmental stages: neonatal samples were generated from newborn mice (0-1day) and adult sample were generated from 4 to 6weeks old animals. This “timestamp mouse” model was generated by crossing TCRδCre-ERT2 (#031679) with ZsGreen (#007906) or TdTomato (#007909) mice, all commercially available at The Jackson Laboratory. Mice were bred in specific pathogen free (SPF) conditions, fed standard chow *ad libitum* and housed under standard temperature, humidity and light/dark cycles. All experiments utilized timed matings to ensure mice were of the required age for each experiment. To activate fluorescent reporter expression in neonatal T cells, 2.5 mgs of tamoxifen were administered to dams by oral gavage three times in 12-h intervals over a 24-h period such that 0-1day old pups received tamoxifen through lactation. To activate fluorescent reporter expression in adult-derived T cells, 1 mg was administered to 4 weeks old mice by oral gavage in 24-h intervals for two days. To assess the properties of recent thymic emigrants, animals were sacrificed at 2–2.5 weeks post-reporter activation. Within experiments, mice were sex-matched, but both sexes were used throughout the study and no association of response with sex was observed or expected.

Mice for this study were maintained at the Cornell College of Veterinary Medicine. The facilities are accredited by the American Association of Accreditation of Laboratory Animal Care. All protocols regarding animal use were reviewed and approved by the Institutional Animal Care and Use Committee at Cornell University (protocol #2011-0090).

### Method details

#### Flow cytometry

For flow cytometry, splenocytes were isolated by manual dissociation and filtration over a 40 μm filter. CD8^+^ T cells were enriched with positive magnetic selection using anti-CD8a microbeads (Miltenyi). Following isolation cells were stained with antibodies purchased from ThermoFisher/Life technologies, BioLegend, or BD Biosciences. For tetramer staining, biotinylated monomer was obtained from the NIH Tetramer core facility and tetramerized with APC-Streptavidin (Life technologies) by sequential titration of streptavidin into monomer stock.

For surface staining only, IC fix buffer set (ThermoFisher/Life technologies) was used. For experiments needing intracellular staining, BD Cytofix/Cytoperm fixation kit was used. Both kits were used according to manufacturer’s instructions. Flow cytofluorimetric data were acquired using FACSDiva software from a BD FACSymphony A3 or A5 SE, both equipped with five lasers (BD Biosciences). Analysis was performed with FlowJo (BD Biosciences).

#### *In vitro* stimulation

CD8^+^ T cells were isolated from the spleen using positive magnetic selection with CD8 microbeads (Miltenyi). Following magnetic bead purification, cells were labeled with Cell Trace Violet (ThermoFisher/Life technologies) according to manufacturer’s recommendation. Cells were stimulated with plate-bound anti-CD3 (2.5 μg/ml, clone 2C11) then cultured with complete RPMI (10% FBS with L-glutamine and Penicillin/Streptomycin) supplemented with 100 U/ml human IL2 and 2 μg/ml anti-CD28 (clone 37.51). Cells were harvested at 48 h, stained for surface markers and then analyzed by flow cytometry.

#### Thymic transplants

Thymic transplants were performed as previously described.^63^ Briefly, thymus lobes were isolated from 0 to 1day old timestamp mice (ZsGreen version). The thymus lobes were separated into individual lobes and were placed under the kidney capsule of an anesthetized 6-week-old timestamp mouse (TdTomato version). To mark thymocytes from both donor and recipient, 5 mg of tamoxifen was administered from 0 to 3days post-transplantation. Recipients were sacrificed at indicated times and spleens were isolated to assess phenotype and behavior of timestamped cells through flow cytometry.

#### Listeria infections

Dr. Sing Sing Way (Cincinnati Children’s Hospital, OH) provided Wild-type *Listeria monocytogenes* expressing epitope HSV-1 gB_498-505_ (Lm-gB).^64^ Bacteria were grown to log phase, and mice were injected intravenously (i.v.) with 5 x 10^3^ colony forming units (CFU) in 100 μl of PBS, as previously described.^10^ At indicated timepoints post-infection, splenocytes were recovered ad processes for flow cytometry.

#### Single-cell RNA-seq

##### scRNA-seq library preparation

Two weeks post timestamp, live, splenic ZsGreen+CD8^+^ cells were FACS sorted on an MA900 cell sorter (Sony) into 0.04% BSA in PBS. Cells were loaded in the Chromium instrument (10X Genomics) for the formation of gel bead-in-emulsions (GEMs) targeting 5-10k cells per sample. Single-cell RNA-seq and TCR libraries were prepared using Chromium Single Cell Universal V(D)J Reagent Kits (v1.1 chemistry; 10X Genomics) by the BRC Genomics Facility following the manufacturer’s protocol. Libraries were sequenced on a NovaSeq 6000 (Illumina) to an average depth of >150M reads (scRNA-seq) and >15M reads (scTCR).

##### scRNA-seq raw data processing and cell quality filtering

Raw FASTQ data was processed using ‘cell ranger multi’ (v6.0.0, 10X Genomics) with provided mouse reference genome refdata-gex-mm10-2020-A and TCR reference refdata-cellranger-vdj-GRCm38-alts-ensembl-5.0.0 for TCRs. The resulting count matrixes were filtered using Seurat v5.0.0 to remove cell barcodes with 1) fewer than 500 genes detected, 2) fewer than 1000 transcripts detected, 3) more than 7.5% mitochondrial counts, and 4) those not passing TCR sequence filtering; only cell barcodes that were assigned a single TRA-TRB pair were retained for analysis. After filtering, we obtained high-quality transcriptomic profiles for 59,281 individual cells with an average of 7,410 cells per sample and 1,560 genes and 4,419 transcripts detected per cell.

##### scRNA-seq dataset preprocessing, identity-based filtering, and clustering

Following cell quality filtering, three rounds of data preprocessing were performed using Seurat v5.0.0. Each preprocessing round included: count normalization, scaling, variance regression, dimensionality reduction, cluster identification, and UMAP generation. Within each preprocessing round, we regressed out variation in mitochondrial read percentage, ribosomal read percentage, cell cycle associated gene expression, and TCR gene segment gene expression. The first 30 dimensions were utilized for cluster identification and UMAP generation in each preprocessing round. Following an initial round of preprocessing, three clusters of cells were removed from the dataset, which corresponded to 1) sample processing stress, 2) dividing cells, and 3) CD8^+^ dendritic cells. After a second round of preprocessing was performed, a small number of cells associated with cell division were again identified; these cells were removed from the dataset. Upon a final round of preprocessing, we identified 11 total clusters (resolution of 0.7) that served as a basis for the downstream analysis.

##### scRNA-seq cluster annotation

Seurat’s FindMarkers function was used to compare each individual cluster of cells to the rest of the dataset, resulting in the identification of the 5 genes that were most upregulated per cluster. Bubble plot visualization of gene expression was generated with the Clustered_DotPlot function of scCustomize v2.1.2.9029^65^. The lower and upper bounds of the bubble plot expression color scale were defined as the 10^th^ percentile expression value and 90^th^ percentile expression value considering the expression of all input genes across all clusters. Gene set module scores were calculated using the AddModuleScore_UCell function of UCell v2.6.2. The true naive and virtual memory gene sets were derived from Smith et al. 2018^14^; ImmGen T cell gene signature gene sets are derived from Best et al. 2014.^43^ GSEA was performed using fgsea v1.28.0^66^ comparing the query gene sets against genes ranked by their fold-change as reported by Seurat’s find markers function. GSEA bubble plots were generated using ggplot2 v3.5.2^67^.

##### scRNA-seq differential expression analysis

For differential expression analysis, we generated pseudo-bulk expression profiles for each cluster by aggregating the raw counts across single cells with the same sample and cluster identity using the aggregate.Matrix command of Matrix.utils v0.9.7^68^. The resulting pseudo-bulk expression profiles were then used as input into DESeq2’s differential expression pipeline.^69^ Genes that possessed ≥3 counts across ≥3 pseudo-bulk samples per cluster were retained for differential expression testing. Age-related differential expression testing was performed within each cluster, and genes with an adjusted *p*-value less than 0.05 were considered to be differentially expressed. Stacked bar plots were generated using ggplot2. GSEA was performed per cluster by comparing query gene sets against genes ranked by their DESeq2 reported fold-changes (neonate vs. adult). Finally, differentially expressed genes were clustered using the K-means clustering algorithm implemented by the Stats package v4.3.2 considering their fold-change values across each cluster.^70^ Six K-means clusters were plotted given the results of plotting the within-cluster sum of squares using the function fvis_nbclust within the factoextra package v1.0.7^71^. Fold-change boxplots were generated using ggplot2. To compare the proportion of neonatal-derived to adult-derived cells within each cluster, the raw counts of cells per sample within each cluster were quantified. Then, the raw counts were normalized for differences in the number of total cells profiled from each sample by dividing each the raw counts by the total number of cells in the corresponding sample, which generated the proportion of cells from each sample contained within each cluster. The resulting normalized proportions were visualized using stacked bar plots generated using ggplot2. To visualize the differences in proportion of neonatal-derived to adult-derived cells within each cluster on the UMAP embedding, the stat_density_2d function (bins = 15, *n* = 200, adjust = 0.9) of ggplot2 was utilized to generate two-dimensional kernel density overlays based on the single-cell UMAP coordinates of either neonatal or adult cells.

##### Integrative scTCR-seq and scRNA-seq analysis

To integrate TCR information with single cell expression profiles, each cell was assigned an annotation of 0, 1–2, or >2 TCRB n-additions. The average proportion of cells with 0, 1–2, and ≥2 N-addition annotations were calculated separately for adult and neonatal cells after first grouping cells by age of origin. Cluster-specific proportions of cells with 0, 1–2, and >2 N-addition annotations were calculated after cells were grouped by their age of origin and gene expression cluster. Differential expression testing was performed between cells with 0 and >2 ≥ 2 N-additions after grouping cells by 1) age of origin, 2) age of origin and virtual memory phenotype (gene expression clusters two and four), and 3) age of origin and true naive phenotype (gene expression clusters zero and three). Gene expression values were calculated for MA plot visualization by aggregating the normalized counts across each cell per gene, for all detected genes. Fold-change values reported were calculated via Seurat’s FindMarkers function. Genes were denoted as significantly differentially expressed if their associated adjusted *p*-values were less than 0.05. Violin plots were generated using normalized count data as input into Seurat’s VlnPlot function. GSEA was performed using fgsea comparing the gene sets against genes ranked by their fold-change as reported by Seurat’s find markers function. GSEA bubble plots were generated using ggplot2, and running enrichment score plots were generated with fgsea’s plotEnrichment function.

#### TCR repertoire analysis

The full-length consensus TCR sequences assembled by Cell Ranger were aligned against the TCR genes using IMGT/HighV-QUEST^72^ to determine the best-matched V, (D), and J genes and the minimum number of nucleotide additions required to generate each TCR sequence. TCR sequences were omitted from subsequent analysis on the basis of non-productive rearrangements, unidentified V or J gene usage, and more than one productive alpha or beta chain. TCR diversity was assessed as the number of unique TCR clonotypes. The diversity analysis was restricted to cells for which there was a TRA-TRB sequence pair and cells were pooled across the 4 mice in each of the neonatal and adult groups. To account for differences in sample size, 12000 cells were randomly drawn from each pooled population and the number of unique TRA, TRB, and TRA-TRB clonotypes were estimated for each of 1000 random draws.

### Quantification and statistical analysis

For flow cytometry experiments, statistical analysis was performed using Prism (Graphpad). Error bars represent SEM. Statistical significance was determined by paired or unpaired Student’s *t* test as indicated in figure legends. Significance for individual figures is denoted in the legend: Ns not significant; ∗∗, *p* < 0.01; ∗∗∗, *p* < 0.001; ∗∗∗∗, *p* < 0.0001.

For genomic analyses, unless explicitly stated in the methods detail section, all quantifications and statistics were performed using default parameters of the indicated software packages.

### Additional resources

The GEO accession number for the sequencing data reported in this paper is GSE288766.
